# Circulating Cytokines and Histological Liver Damage in Chronic Hepatitis B Infection

**DOI:** 10.1155/2013/757246

**Published:** 2013-10-31

**Authors:** Kittiyod Poovorawan, Pisit Tangkijvanich, Chintana Chirathaworn, Naruemon Wisedopas, Sombat Treeprasertsuk, Piyawat Komolmit, Yong Poovorawan

**Affiliations:** ^1^Division of Gastroenterology, Department of Medicine, Faculty of Medicine, Chulalongkorn University, Bangkok 10330, Thailand; ^2^Department of Biochemistry, Faculty of Medicine, Chulalongkorn University, Bangkok 10330, Thailand; ^3^Department of Microbiology, Faculty of Medicine, Chulalongkorn University, Bangkok 10330, Thailand; ^4^Department of Pathology, Faculty of Medicine, Chulalongkorn University, Bangkok 10330, Thailand; ^5^Department of Pediatrics, Center of Excellence in Clinical Virology, Faculty of Medicine, Chulalongkorn University, Bangkok 10330, Thailand

## Abstract

Each phase of hepatitis B infection stimulates distinct viral kinetics and host immune responses resulting in liver damage and hepatic fibrosis. Our objective has been to correlate host inflammatory immune response including circulating Th1 and Th2 cytokines in patients with chronic hepatitis B infection with liver histopathology. Sixty-four patients with chronic hepatitis B without previous treatment were recruited. The liver histology and histological activity index were assessed for various degrees of necroinflammation and hepatic fibrosis. We determined circulating levels of the Th1 and Th2 cytokines. Forty-six males and 18 females at a median age of 34.5 years were studied. HBeAg was present in 28/64 (43.75%) of the patients. In patients negative for HBeAg, IL-10 and IFN-gamma were significantly correlated with degrees of necroinflammation (*r* = 0.34, *r* = 0.38, resp.; *P* < 0.05). Moreover, TNF-alpha was significantly correlated with degrees of fibrosis (*r* = 0.35; *P* < 0.05), and IL-10 and TNF-alpha were significantly correlated with significant fibrosis (*r* = 0.39, *r* = 0.35, resp.; *P* < 0.05). These correlations were found in the HBeAg negative group as opposed to the HBeAg positive group. In HBeAg negative patients, circulating cytokines IL-10 and IFN-gamma were correlated with degrees of necroinflammation, whereas IL-10 and TNF-alpha were correlated with significant fibrosis.

## 1. Introduction

Chronic hepatitis B infection is a major cause of chronic liver disease worldwide. Each phase of hepatitis B infection stimulates distinct viral kinetics and host immune responses resulting in liver damage and hepatic fibrosis [1]. Vaccine induced immune response in humans has provided excellent long term protection and result decline prevalence of hepatitis B virus (HBV) infection in long term [2, 3]. In contrast to host immune response in chronic hepatitis B infection, due to immune tolerance, only a small proportion of chronic hepatitis B (CHB) patients could clear the infection [4]. Histological damage and risk of HCC in CHB patients depend on various parameters such as duration of infection, coinfection with other hepatitis viruses, and alcohol consumption [5]. Cytokines are known to play a significant role in host immune responses. In chronic hepatitis B patients, the concentration of circulating Th17 cells (producing IL-17) increased with disease progression from CHB (mean, 4.34%) to acute-on-chronic liver failure (mean, 5.62%) patients as compared to healthy controls (mean, 2.42%) [6]. Furthermore, higher serum levels of IL-10 and IL-12 in HBeAg positive patients are correlated with early, spontaneous HBeAg seroconversion [7]. T cells play a major role in the immunopathogenesis associated with chronic hepatitis B. T cells destroy infected hepatocytes and suppress HBV replication [8]. Our objective has been to correlate host inflammatory immune response including circulating Th1 and Th2 cytokines in patients with chronic hepatitis B infection with the liver histopathology observed. 

## 2. Materials and Methods

A cross-sectional prospective study was conducted on chronic HBV patients who were evaluated for treatment at Chulalongkorn King Memorial Hospital from 2010 to 2012. The research protocol was approved by the Institutional Review Board (IRB number 515/53) of the Faculty of Medicine, Chulalongkorn University. The objective of the study was explained to the patients, and subsequently, written consent was obtained.

### 2.1. Patients

Sixty-four patients with chronic hepatitis B without previous treatment were recruited. Clinical, demographic, and laboratory data were collected. Patients with evidence of HCV or HIV coinfection, alcoholic liver disease, and chronic liver disease due to other causes and acute viral hepatitis B were excluded from the study. 

### 2.2. Specimen Collection

From January 2010 to January 2012, 64 samples were collected from patients with chronic HBV infection. Samples were collected as clotted blood and sera were separated within 6 hours. All specimens were kept at −70°C until tested.

### 2.3. Clinical Assessment

Based on their HBeAg status, patients were classified into an HBeAg positive and an HBeAg negative group. Liver biopsy was performed by percutaneous needle biopsy (16-gauge, Menghini). Specimen length of at least 1.5 cm and at least 10 portal tracks is required for an adequate evaluation [9]. 

Histology and histological activity index (HAI) were graded according to degrees of necroinflammation applying the following score: 0 = no inflammation, 1–4 = minimal inflammation, 5–8 = mild inflammation, 9–12 = moderate inflammation, and 13–18 = marked inflammation as described by Knodell et al. [10]. Hepatic fibrosis was staged on a 5-point scale (F0: no fibrosis; F1: minimal fibrosis; F2: fibrosis with a few septa; F3: numerous bridging fibroses without cirrhosis; F4: cirrhosis or advanced severe fibrosis) as described in the Metavir score. F0-F1 was defined as nonsignificant fibrosis and F2–F4 as significant fibrosis. 

### 2.4. Liver Stiffness Measurement

Transient elastography was performed by a well-trained nurse using FibroScan 502 (Echosens, Paris, France). The median value of 10 validated scores was considered the elastic modulus of the liver, and it was expressed in kilopascals (kPa).

### 2.5. Laboratory Method

Circulating levels of Th1 cytokines comprising IL-2, IL-12p70, and interferon-gamma (IFN-gamma) and Th2 cytokines including IL-4, IL-5, IL-10, IL-13, and also TNF-alpha and GMCSF were measured by ELISA (Bio-Plex Cytokine Assays). We quantitatively determined the HBsAg titer by ELISA method (Elecsys, Roche Diagnostics, Indianapolis, IN, USA), and the HBV DNA concentration was measured by quantitative real time PCR (The Abbott m2000sp Real Time System). 

We determined serum anti-HCV and anti-HIV by enzyme immunoassay (Architect, Abbott Diagnostics, Germany).

### 2.6. Statistical Analysis

Continuous variables were compared between groups using unpaired *t*-test and one-way ANOVA. Categorical variables were compared between groups using chi-square/Fisher's exact test. Pearson's correlation coefficient was used to describe the correlation between two continuous, normally distributed variables. Spearman's correlation was used where variables were not normally distributed. All statistical analyses were performed using SPSS version 16.

## 3. Results

Forty-six males and 18 females at a median age of 34.5 years were studied. HBeAg was present in 28 of 64 (43.75%) patients. In the study population, HBeAg negative patients were older than those positive for HBeAg. HBV DNA, HBsAg titer, and ALT concentrations were significantly higher in the HBeAg positive group. Degrees of hepatic necroinflammation, liver fibrosis, and liver stiffness measured by FibroScan were not significantly different between both groups (Table 1).

Among all patients, cytokine levels were not correlated with ALT, DNA levels, and liver stiffness measurement by FibroScan. The correlation between IL-5 and HBsAg titer was negative (*r* = −0.239; *P* < 0.05).

In HBeAg negative patients, inflammatory cytokine IL-5 and IL-12p70 levels were significantly higher than in HBeAg positive patients (0.726 versus 0.508 and 2.17 versus 0.217, resp.; *P* < 0.05). Other mean serum cytokine levels were not statistically different (Table 2).

**Figure 1:**
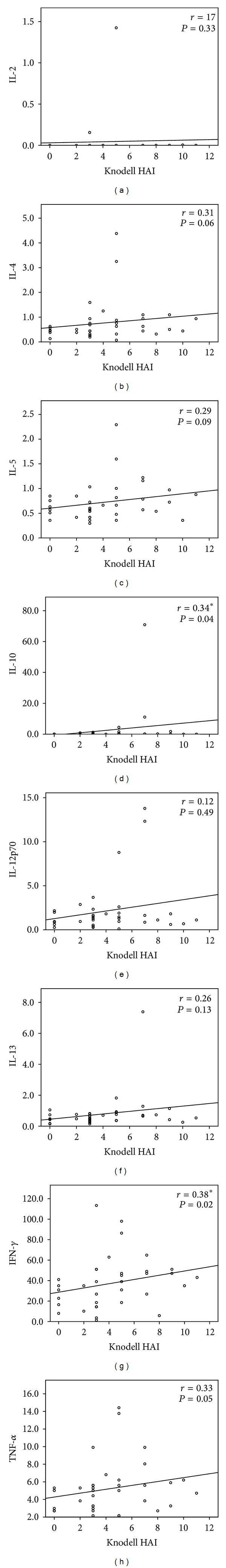
Correlation between circulating cytokine levels (pg/mL) and degrees of necroinflammation (Knodell histological activity index) in the HBeAg negative group (*N* = 36). ^†^Spearman's rank correlation coefficient, *statistically significant.

**Figure 2:**
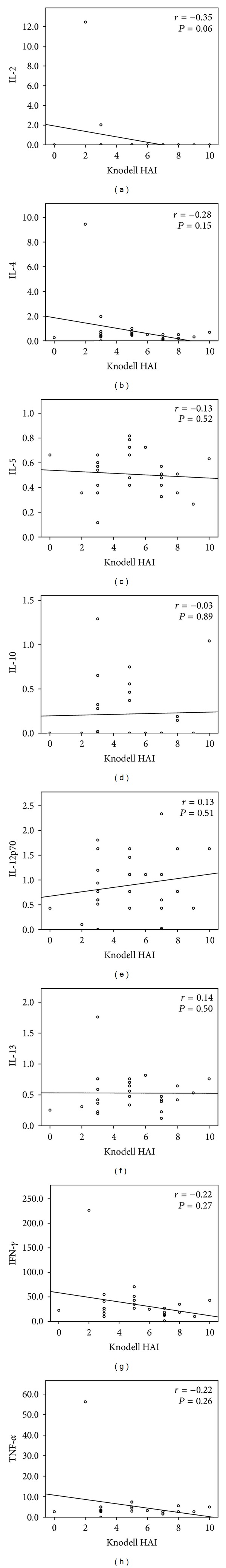
Correlation between circulating cytokine levels (pg/mL) and degrees of necroinflammation (Knodell histological activity index) in the HBeAg positive group (*N* = 28). ^†^Spearman's rank correlation coefficient.

In the HBeAg negative group, IL-10 and IFN-gamma were significantly correlated with degrees of necroinflammation (*r* = 0.336, *r* = 0.380, resp.; *P* < 0.05). while other cytokines were not correlated (Figure 1). In the HBeAg positive group, none of the cytokines was correlated with degrees of necroinflammation (Figure 2). After multivariate analysis with baseline characteristic, laboratory data, and other cytokines, IL-10 was the only one parameter which significantly correlated with degrees of necroinflammation (Table 3).

TNF-alpha was significantly correlated with degrees of fibrosis (*r* = 0.35; *P* < 0.05), and IL-10 and TNF-alpha were significantly correlated with significant fibrosis in HBeAg negative patients (*r* = 0.39, *r* = 0.35, resp.; *P* < 0.05). After multivariate analysis with baseline characteristic, associated laboratory data and other cytokines, there was no significant correlation between IL-10, TNF-alpha, and significant fibrosis. In the HBeAg positive group, none of the cytokines was correlated with fibrosis.

## 4. Discussion 

Chronic hepatitis B infection results in a complex interplay between virus and host immune response. T-cell immune response is correlated with fibrosis and hepatic inflammation in HBV chronic hepatitis and cirrhotic patients [11]. The phase of disease is determined by clinical characteristics of liver inflammation and virus replication in the host. Chronic hepatitis B (CHB) infection progresses through various phases in relation to the host, that is, immune tolerant, immune active, and inactive carrier. The immune response in each phase of disease depends on complex mechanisms. Recent data have demonstrated that even the immune tolerant phase of CHB is not associated with an immune profile of T-cell tolerance [12].

 Cytokines contribute to the immune response and have a specific response to each disease [13]. T-helper cytokines in CHB patients and healthy controls were different. For example, serum IL-33 was significantly higher in healthy controls at the baseline but decreased after antiviral treatment [14]. Some data have demonstrated that the circulating cytokine profile in chronic hepatitis B is related to the HBeAg status, virus replication, and stage of liver disease [15, 16]. 

 Inflammatory cytokine IL-5 and IL-12p70 levels were significantly higher in the HBeAg negative group. A previous study has demonstrated that IL-5 as well as IL-4 levels were increased in acute self-limited hepatitis B [17]. IL-12 was found to significantly augment the HBcAg-specific secretion of IFN-gamma in CHB children and, thus, to increase the probability of HBeAg seroconversion in CHB patients [7, 18]. Treatment of CHB patients with a combined regimen of IL-12 and lamivudine enhanced T-cell reactivity to HBV and IFN-gamma production. However, IL-12 did not suppress HBV replication in HBeAg positive patients and did not uphold inhibition of HBV replication after lamivudine withdrawal [19].

 Interleukin-10 (IL-10) generally suppresses cellular immune responses by modulating the function of T cells and antigen-presenting cells [20]. In contrast to CHB patients, IL-10 was related to the HBeAg status, virus replication, and liver disease progression [16]. This study confirmed the correlation between IL-10 and histological liver damage in HBeAg negative CHB patients. Furthermore, a recent study has demonstrated that the IL-10 level can also serve as a predictor of HBeAg seroconversion in CHB patients [7]. 

 IFN-gamma and TNF-alpha were thought to be important immune mediators in the host defense against hepatitis B virus (HBV) infection. These cytokines can induce apoptosis in liver cells expressing HBV [21]. TNF-alpha mediates an innate antiviral response that targets the integrity of HBV nucleocapsids [22]. IFN-gamma suppresses hepatitis B virus replication and significantly reduces expression of the large HBV surface protein (LHBs) and hepatocytes' microscopical appearance of ground glass [23].

This study has established that circulating cytokines IL-10 and IFN-gamma are correlated with degrees of necroinflammation in HBeAg negative patients, whereas IL-10 and TNF-alpha are correlated with significant fibrosis. These correlations were found in the HBeAg negative group as opposed to the HBeAg positive group. However, further research should be performed to determine the exact roles of each cytokine in liver necroinflammation and fibrogenesis.

## Figures and Tables

**Table 1:** Comparison of demographic, clinical, and ALT level, histopathology, and liver stiffness characteristics between HBeAg positive and HBeAg negative patients*.

	HBeAg positive (*n* = 28)	HBeAg negative (*n* = 36)	*P* value
Age > 40yrs	3/28 (10.7%)	21/36 (58.3%)	<0.01
Age (yrs)	30.9 ± 7.9	42 ± 10.2	0.02
Male gender	17/28 (60.7%)	28/36 (77.8%)	0.14
Mean BMI	25.1 ± 4.8	23.7 ± 3.8	0.43
Underlying of DM	0	2/36 (5.6%)	0.27
ALT > 60 U/L	20/28 (71.4%)	16/36 (44.4%)	0.03
HBV DNA (logIU/mL)	7.08 ± 1.39	5.34 ± 1.35	<0.01
HBsAg titer (IU/mL)	20380.8 ± 21308	5477 ± 7557.3	<0.01
Knodell HAI ≥ 4	17/28 (60.7%)	17/36 (47.2%)	0.28
Significant fibrosis	11/28 (39.3%)	21/36 (58.3%)	0.13
Liver stiffness (Kpa)	7.13 ± 2.49	7.76 ± 3.72	0.09

*Plus-minus values are means ± SD for all comparisons.

**Table 2:** Comparison of cytokine levels between HBeAg positive and HBeAg negative patients*.

(pg/mL)	HBeAg positive (*n* = 28)	HBeAg negative (*n* = 36)	*P* value
IL-2	0.517 ± 2.37	0.726 ± 0.38	0.24
IL-4	0.79 ± 1.74	0.77 ± 0.83	0.95
IL-5	0.508 ± 0.16	0.726 ± 0.38	<0.01**
IL-10	0.217 ± 0.35	2.607 ± 11.9	0.29
IL-12p70	0.898 ± 0.60	2.17 ± 3.06	0.03**
IL-13	0.529 ± 0.31	0.803 ± 1.18	0.24
GMCSF	1.131 ± 0.1.75	1.56 ± 1.84	0.35
IFN-gamma	34.8 ± 40.72	37.36 ± 25.43	0.76
TNF-alpha	5.446 ± 10.09	5.196 ± 2.93	0.89

*Plus-minus values are means ± SD for all comparisons.

**Statistically significant.

**Table 3:** Multivariate analyses of the association between degrees of necroinflammation and other parameters.

Parameters	Standardized coefficients	*P* value
Age	−0.13	0.51
BMI	0.02	0.93
ALT	0.38	0.13
HBV DNA	0.07	0.82
HBsAg titer	−0.16	0.6
IL-2	−0.38	0.19
IL-4	0.35	0.51
IL-5	0.16	0.66
IL-10	0.96	0.04*
IL-12p70	−1.69	0.06
IL-13	0.67	0.16
GMCSF	−0.70	0.18
IFN-gamma	0.27	0.48
TNF-alpha	0.76	0.09

*Statistically significant.
